# Implementation of a specialized neuroprognostication consultation program and associated provider attitudes: A survey-based study

**DOI:** 10.1016/j.resplu.2025.100932

**Published:** 2025-03-19

**Authors:** David Fischer, Sahily Reyes-Esteves, Connor Law, Alice Ford, Peter Schwab, Benjamin S. Abella, Andrea L.C. Schneider, Monisha A. Kumar

**Affiliations:** a Department of Neurology, Perelman School of Medicine, University of Pennsylvania, PA, USA; b Department of Emergency Medicine, Icahn School of Medicine at Mount Sinai, NY, USA; c Department of Biostatistics, Epidemiology, and Informatics, Perelman School of Medicine, University of Pennsylvania, PA, USA

**Keywords:** Cardiac arrest, Neuroprognostication, Disorders of consciousness

## Abstract

**Introduction:**

Neuroprognostication for disorders of consciousness, particularly after cardiac arrest, is critical. However rapidly evolving research has translated little to clinical practice, with neuroprognostication frequently deviating from evidence and clinical guidelines. We implemented a novel program that provides specialized, interdisciplinary, and longitudinal care to improve the practice of neuroprognostication. The objective of this study was to evaluate the impact of this program on provider attitudes and satisfaction towards neuroprognostication after cardiac arrest.

**Methods:**

We disseminated surveys across our health system to critical care providers and neurologists in the years before (2021, 2022) and after (2023) implementation of the program. The surveys assessed perceptions of, and satisfaction with, neuroprognostication after cardiac arrest. We used Fisher exact tests to compare program-exposed respondents to historical controls (2021 and 2022 respondents) and contemporary controls (2023 respondents without program exposure).

**Results:**

We received 545 responses from neurologists and critical care providers, including nurses, trainees, and attendings. Program-exposed respondents, relative to historical and contemporary controls respectively, reported greater usefulness of neuroprognostication (94% reporting often or always useful, versus 69% [*p* < 0.01]) and 68% [*p* < 0.01]), comprehensiveness of neuroprognostication (94% reporting often or always comprehensive, versus 76% [*p* = 0.02] and 66% [*p* < 0.01]), and greater satisfaction with neuroprognostication, particularly in comparison to the conventional model (63% reporting the program was “much better”).

**Conclusion:**

Implementation of a specialized neuroprognostication program was associated with largely favorable attitudes towards neuroprognostication among providers. These findings encourage further study of this paradigm, and consideration of broader adoption to improve the practice of neuroprognostication.

## Highlight

• Does a specialized neuroprognostication program improve provider perceptions?
• We disseminated surveys to critical care providers over three years.
• Surveys assessed attitudes and satisfaction with post-arrest neuroprognostication.
• The program was associated with improved provider attitudes and satisfaction.
• A specialized program may help improve neuroprognostication after cardiac arrest.

## Graphical abstract

**Figure:**
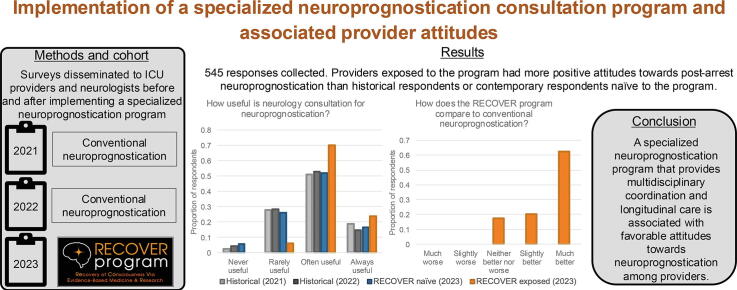

## Introduction

Neuroprognostication after acute brain injury, such as hypoxic-ischemic brain injury from cardiac arrest, is impactful, frequently influencing decisions to withdraw life-sustaining treatments.^1^ A growing literature aims to improve the accuracy of prognostic biomarkers.^2^ However, there is a widening gap between the advances of research and the practice of neuroprognostication.^3^ Clinical neuroprognostication frequently deviates from evidence and guidelines,^4^, ^5^ and studies estimate that some patients may have life-sustaining treatment withdrawn prematurely.^6^, ^7^

To address this gap, we established the Recovery of Consciousness Via Evidence-Based Medicine and Research (RECOVER) program to provide a specialized, comprehensive, and longitudinal approach to neuroprognostication.^8^ This novel paradigm, detailed elsewhere,^8^ includes a dedicated neuroprognostication consult service involving a neurology-led multidisciplinary team of expert clinicians that convene through regular conferences (e.g., neuroradiologists, epileptologists, physiatrists, palliative care specialists, physical/occupational therapists); offers continuity through the acute, post-acute, and chronic phases of recovery; promotes trainee education; and integrates research to facilitate data collection and translation. The impact of this program has not yet been studied. It is uncertain, for example, whether providers will find this specialized and multidisciplinary approach to be helpful or burdensome.

We used surveys to assess provider attitudes towards neuroprognostication after cardiac arrest (the majority of neuroprognostication cases at our center^8^) before and after RECOVER program implementation. We hypothesized that implementation of this program would be associated with favorable provider attitudes and satisfaction towards post-arrest neuroprognostication.

## Methods

We conducted this study at three academic tertiary hospitals within Penn Medicine: one where the RECOVER program was implemented (Hospital of the University of Pennsylvania [HUP]), and two others where it was not (Penn Presbyterian Medical Center, and Pennsylvania Hospital). We developed an internet-based, anonymized survey (REDCap, Nashville, TN) 9, 10 including 35 questions, 20 of which pertained to provider attitudes and satisfaction about neuroprognostication and were the focus of this study (Supplementary Methods). In the absence of existing validated scales for this context, we formulated these questions to capture the attitudes that might define the value of neuroprognostication, including how providers perceive the usefulness and comprehensiveness of neuroprognostication, how indiscriminately neuroprognostication consultation leads to the withdrawal of life-sustaining treatment, and the education providers receive about neuroprognostication. To elicit additional opinions, we prompted RECOVER exposed respondents to provide positive and negative free-text feedback about the program. Though a formal thematic analysis was outside the scope of this study, we organized responses by topic. We distributed the survey twice prior to the implementation of the RECOVER program in 8/2022 (6/2021 and 8/2022, to evaluate temporal trends preceding program implementation) and once following (10/2023). The surveys targeted intensive care unit (ICU) providers (attendings, advanced practice practitioners, fellows, residents and nurses) and neurologists via departmental emails and flyers. No financial incentives were offered. To prioritize anonymity, we did not collect identifying information; it is thus uncertain what proportion of respondents were maintained across surveys. This study was approved and deemed exempt from informed consent by the University of Pennsylvania Institutional Review Board (#857143 on 10/31/24).

Respondent characteristics are shown using numbers and proportions. Surveys were categorized by distribution year: two prior to program implementation (2021 and 2022) and one after (2023). 2023 survey respondents were divided into those who, when asked whether they had interfaced with the RECOVER program in providing clinical care (Supplementary Methods), responded “yes” (RECOVER exposed) versus “no” (RECOVER naïve). To evaluate program-associated attitude differences, we used Fisher exact tests to compare RECOVER exposed respondents to contemporary controls (RECOVER naïve respondents), and historical controls (2021 and 2022 respondents). Statistical significance was set *a priori* as a two-sided alpha of 0.05. To adjust for changes in respondent characteristics between surveys (e.g., proportions of trainee and non-trainee physicians), we conducted stratified analyses, where relevant, to facilitate comparisons between surveys. Analyses were performed using R version 4.4.1 (R Foundation for Statistical Computing; Vienna, Austria). Anonymized data are available by request from any qualified investigator.

## Results

**Fig. 1:**
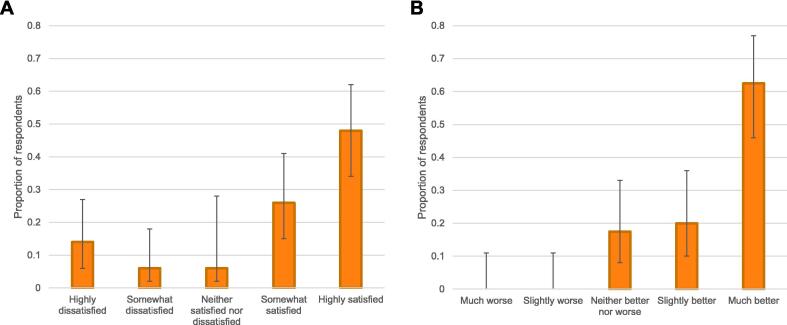
**RECOVER Program Satisfaction.** Both panels illustrate responses from RECOVER exposed respondents. Panel (A) illustrates responses to “How satisfied were you with the RECOVER service?” Panel (B) illustrates responses to “How do you feel the RECOVER service (launched at HUP in August 2022) compares to conventional neurology consultations for neuroprognostication?”, among the 80% of RECOVER exposed respondents who felt they had sufficient experience to evaluate (20% of RECOVER exposed respondents reported insufficient experience). The Y axes of all panels represent the proportion of respondents within each group who provided each response. Error bars represent 95% confidence intervals.

There were 545 responses across the 2021, 2022, and 2023 surveys (Table 1). RECOVER exposed respondents reported satisfaction with the program (mean 4 of 5). The greatest proportion reported being “highly satisfied” (Fig. 1A). Among the 80% of RECOVER exposed respondents who could compare to the prior conventional neuroprognostication model (where neuroprognostication was provided by a general neurology consultation service), 63% (95% confidence intervals [CI] = 0.46, 0.77) reported that the RECOVER program was “much better” (Fig. 1B; Supplementary Table 1). Free-text feedback about the program is summarized in Table 2.

**Table 1:** Respondent demographics.

	**2021 survey**	**2022 survey**	**2023 survey: Total**	**2023 survey: RECOVER naïve**	**2023 survey: RECOVER exposed**
**Number of respondents**	299	123	123	73	50
**Role on patient care team**					
Attending physician	74 (24.7%)	41 (33.3%)	50 (40.7%)	28 (38.4%)	22 (44.0%)
Trainee (resident or fellow)	60 (20.1%)	23 (18.7%)	32 (26.0%)	7 (9.6%)	25 (50.0%)
Advanced practice provider	36 (12.0%)	17 (13.8%)	6 (4.9%)	6 (8.2%)	0 (0%)
Nurse	129 (43.1%)	42 (34.1%)	35 (28.5%)	32 (43.8%)	3 (6.0%)
**Specialty**					
Anesthesia	11 (3.7%)	6 (4.9%)	6 (4.9%)	2 (2.7%)	4 (8.0%)
Surgery	32 (10.7%)	18 (14.6%)	6 (4.9%)	6 (8.2%)	0 (0%)
Medicine	73 (24.4%)	21 (17.1%)	36 (29.3%)	15 (20.5%)	21 (42.0%)
Neurology	54 (18.1%)	36 (29.3%)	40 (32.5%)	18 (24.7%)	22 (44.0%)
Nursing	129 (43.1%)	42 (34.1%)	35 (28.5%)	32 (43.8%)	3 (6.0%)
**Years in practice**					
Resident or fellow	60 (20.1%)	23 (18.7%)	33 (26.8%)	8 (11.0%)	25 (50.0%)
Attending: 0–5 years	84 (28.1%)	36 (29.3%)	36 (29.3%)	26 (35.6%)	10 (20.0%)
Attending: 6–10 years	62 (20.7%)	17 (13.8%)	22 (17.9%)	18 (24.7%)	4 (8.0%)
Attending: >10 years	93 (31.1%)	47 (38.2%)	32 (26.0%)	21 (28.8%)	11 (22.0%)
**Post-survey respondents who completed pre-survey**	N/A	N/A	33 (26.8%)	19 (26.0%)	14 (28.0%)

**Table 2:** Summary of positive and negative feedback.

**Topic**		**Example comments**
Positive	Improvements upon conventional neuroprognostication	- “I appreciate having an additional expert perspective on patient prognosis and management…” - Neuroprognostication previously performed “without strong neurology engagement” - “It has helped with the load on the general consult service.”
	Communication	- “Help with communicating prognosis with families” - “Very responsive… spend a lot of time with families” - “Family meetings have also been more smooth with this new service.”
	Standardization	- “Brings consistent expertise to the patient's bedside and provides a consistent and detailed multidisciplinary assessment of neuroprognostication…. I believe this is the gold standard of patient care.” - “Systematic and consistent approach to patients” - “Protocolized approach; data-driven; less fear of underestimating chance of recovery”
	Continuity	- Looks “beyond the short-term care/management of patients.”
	Multidisciplinary coordination	- “Provides a thoughtful, well-run, multidisciplinary weekly case conference…. It brings together disparate resources in an organized fashion to serve the patient”
	Education	- “Excellent learning and practice with comatose examination.” - “Educational for both the clinicians and families”
	Integration of research	- “It collects data for further research on neuroprognostication”
Negative	Logistics	- “It took a long time to get recommendations” - “Can cause conflicts with the general consult team”
	Redundancy	- “Seems redundant but adds cost, inefficiency” - “Diversion of clinical resources that are already stretched thin.”
	Perception of bias	- “The RECOVER team is almost uniformly encouraging teams and families not to withdraw care”
	Integration of research	- “A service that is a hybrid research-clinical entity is ethically fraught and the two endeavors should be separated to protect the integrity of both data and patient care.” - “As data become available, would be nice to see the longer term results of patients engaged in recover” - “Sometimes timing/presence of RECOVER study instruments (surveys, imaging, etc) … can cause disruptions.”

**Fig. 2:**
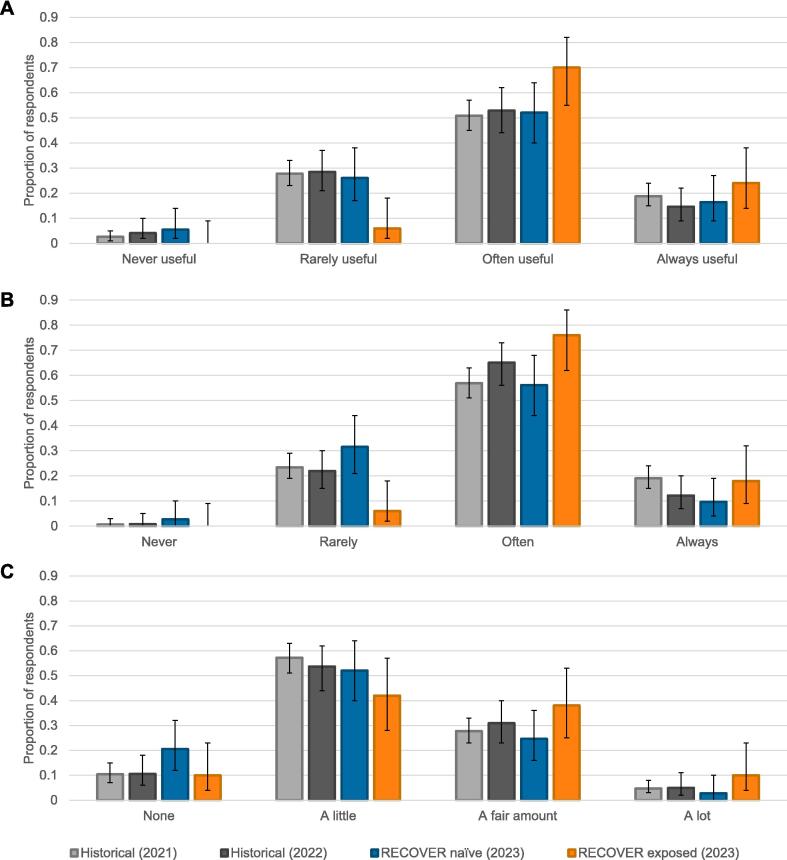
**Attitudes towards neuroprognostication before and after RECOVER program implementation.** For all panels, respondents are divided into four groups: respondents to the 2021 and 2022 surveys (historical controls, combined for statistical analyses), respondents to the 2023 survey who reported exposure to the RECOVER program (RECOVER exposed) and respondents to the 2023 survey who denied exposure to the RECOVER program (RECOVER naïve [contemporary controls]). The Y axes of all panels represent the proportion of respondents within each group who provided each response. Error bars represent 95% confidence intervals. Panel (A) illustrates responses to “How useful is neurology consultation with respect to neuroprognostication in comatose post-cardiac arrest patients?”, panel (B) illustrates responses to “In your experience, how often is a comprehensive neuroprognostic assessment performed on comatose post-cardiac arrest patients?”, and panel (C) illustrates responses to “How much education have you received regarding neuroprognostication in comatose post-cardiac arrest patients?”. (For interpretation of the references to colour in this figure legend, the reader is referred to the web version of this article.)

Implementation of the RECOVER program was associated with improved attitudes towards the utility of neurologic consultation for neuroprognostication. When asked, “*How useful is neurology consultation with respect to neuroprognostication in comatose post-cardiac arrest patients?*” RECOVER exposed respondents reported greater usefulness (94% reporting often or always useful, [95% CI = 0.82, 0.98]) than historical (69% [95% CI = 0.64, 0.73], *p* < 0.01) and contemporary controls (68% [95% CI = 0.56, 0.79], *p* < 0.01) (Fig. 2A; Supplementary Table 2). When asked, “*Do you agree with the following statement? 'There is such limited evidence for the tests used in neuroprognostication that I do not see value in a neurology consult for neuroprognostication'”*, differences did not reach statistical significance (Supplementary Fig. 1). To evaluate associated changes in consultative practices, we asked, “*In your experience, roughly how often is neurology consulted regarding neuroprognostication in comatose post-cardiac arrest patients?*”. RECOVER exposed respondents reported a higher frequency of neurology consultation (98% reporting often or always [95% CI = 0.88, 1.00]) than historical (84% [95% CI = 0.80, 0.87], *p* = 0.02) and contemporary controls (84% [95% CI = 0.73, 0.91], *p* = 0.02) (Supplementary Fig. 2).

Implementation of the RECOVER program was associated with a perceived increase in neuroprognostication comprehensiveness. When asked, “*In your experience, how often is a comprehensive neuroprognostic assessment performed on comatose post-cardiac arrest patients?*” RECOVER exposed respondents reported greater frequencies (94% reporting often or always [95% CI = 0.82, 0.98]) than historical (76% [95% CI = 0.72, 0.80], *p* = 0.02) and contemporary controls (66% [95% CI = 0.54, 0.76], *p* < 0.01) (Fig. 2B; Supplementary Table 3).

Free-text feedback elicited a concern that the RECOVER program may indiscriminately promote continuation of life-sustaining treatment (Table 2). However, when asked, “*Do you agree with the following statement? 'Neurology consultation for neuroprognostication often leads to withdrawal of life-sustaining support in comatose post-cardiac arrest patients.'*” there was no significant difference in these perceptions between RECOVER exposed respondents (64% reporting somewhat or strongly disagree [95% CI = 0.49, 0.77]) and historical (55% [95% CI = 0.50, 0.60]) or contemporary (60% [95% CI = 0.48, 0.71]) controls (Supplementary Fig. 3).

When asked, “*How much education have you received regarding neuroprognostication in comatose post-cardiac arrest patients?*” there were non-significant differences between RECOVER exposed respondents (48% reporting a fair amount or a lot [95% CI = 0.34, 0.62]) and historical (27% [95% CI = 0.18, 0.39], *p* = 0.13) and contemporary controls (33% [95% CI = 0.29, 0.38], *p =* 0.07) (Fig. 2C). We repeated the analysis excluding trainees (n = 115 excluded), after which RECOVER exposed respondents (56% [95% CI = 0.35, 0.75]) reported significantly more education than contemporary controls (29% [95% CI = 0.19, 0.41], *p* = 0.04); differences with historical controls did not reach statistical significance (36% [95% CI = 0.31, 0.41], *p* = 0.08) (Supplementary Fig. 4). When asked, “*How comfortable do you feel providing a neuroprognostic assessment for comatose patients after cardiac arrest?*” we found no significant differences (Supplementary Fig. 5). To evaluate changes in knowledge, we asked respondents about guideline-based practices. When asked, “*In comatose post-cardiac arrest patients who undergo targeted temperature management, when is neuroprognostication ideally performed?*” the greatest proportion of respondents (47–56%) correctly identified 72 h post-rewarming as per guidelines^11^, ^12^, ^13^ (versus earlier), with no significant difference in RECOVER exposed respondents (Supplementary Fig. 6).

## Discussion

We found an association between RECOVER program implementation and favorable attitudes towards neuroprognostication. Though some effects were stronger than others, as a whole these results confirmed our initial hypotheses. RECOVER exposed respondents, relative to others, reported greater usefulness and comprehensiveness of neuroprognostication, more frequent neurology consultations for neuroprognostication, and possibly more neuroprognostication education. Most RECOVER exposed respondents also reported high satisfaction with the program, particularly compared to the conventional model. Notably, 43% of respondents who reported being “highly dissatisfied” with the program also reported that the program was “much better” than conventional neuroprognostication and provided only positive free-text feedback, suggesting possible response error.

Regarding limitations, this associational study cannot confirm causality between implementation of the RECOVER program and these subsequent changes. This study could not assess differences within individuals across time, and RECOVER exposure may be non-random (e.g., those with favorable attitudes towards neuroprognostication may preferentially seek RECOVER consultation). Providers with stronger attitudes may be more likely to complete the survey, leading to response bias. Respondent characteristics changed over time and varied between those exposed and naïve to the RECOVER program, which may introduce confounding, though patterns typically persisted after accounting for these differences in stratified analyses. The RECOVER program is multifaceted, and thus it is uncertain which aspects of the program may have driven these attitude differences. The free-text responses identified specialization, standardization, family meeting assistance, continuity, and multidisciplinary conferences as strengths, but further work is necessary to pinpoint the most impactful elements.

There are several other potential future directions, such as determining how these attitudes translate to patient care. Randomizing exposure to the RECOVER program, perhaps most feasible at institutions newly adopting this paradigm, may better control for confounding. Investigating the impact of the RECOVER program on research and clinical outcomes will be critical. While we note that the RECOVER program primarily coordinates existing resources (rather than mandating new ones), evaluating the program’s cost-effectiveness across different healthcare settings is also important.

## Conclusions

Neuroprognostication after cardiac arrest, though critical, is often hampered by variability and poor coordination of resources. These findings provide evidence that the specialized and multidisciplinary RECOVER program is associated with favorable provider attitudes towards neuroprognostication, encouraging further study to help inform whether and how this novel model of neuroprognostication should be more broadly adopted.

## Conflicts of interest

D.F. is the Director of the RECOVER program. B.S.A. has received funding and consulting honoraria from Becton Dickinson.

## CRediT authorship contribution statement

**David Fischer:** Writing – original draft, Visualization, Supervision, Conceptualization. **Sahily Reyes-Esteves:** Writing – review & editing, Methodology, Investigation, Conceptualization. **Connor Law:** Writing – review & editing, Software, Formal analysis. **Alice Ford:** Investigation. **Peter Schwab:** Investigation. **Benjamin S. Abella:** Writing – review & editing, Supervision. **Andrea L.C. Schneider:** Writing – review & editing, Supervision, Methodology, Formal analysis. **Monisha A. Kumar:** Writing – review & editing, Supervision, Conceptualization.

## Declaration of competing interest

The authors declare that they have no known competing financial interests or personal relationships that could have appeared to influence the work reported in this paper.
