# Supplementary material for: Implementation of a specialized neuroprognostication consultation program and associated provider attitudes: A survey-based study
Source: Resusc Plus. 2025 Mar 19;23:100932. doi: 10.1016/j.resplu.2025.100932 (PMC11999623; doi:10.1016/j.resplu.2025.100932)
Supplement: Supplementary Data 1 [file mmc1.docx]

**Supplementary Table 1. Satisfaction with RECOVER program among RECOVER exposed respondents**

|  | **Overall (50)** | **Neurologist physicians (9)** | **Non-neurologist physicians (13)** | **Nurses (3)** | **Residents and fellows (25)** | **Advanced practice providers (0)** |
| --- | --- | --- | --- | --- | --- | --- |
| **Satisfaction with RECOVER service (mean)** | 3.88/5 | 3.22/5 | 4.23/5 | 3/5 | 4.04/5 | N/A |
| Highly dissatisfied (1) | 7 | 2 | 2 | 1 | 2 | N/A |
| Somewhat dissatisfied (2) | 3 | 2 | 0 | 0 | 1 | N/A |
| Neither satisfied nor dissatisfied (3) | 3 | 0 | 0 | 1 | 2 | N/A |
| Somewhat satisfied (4) | 13 | 2 | 2 | 0 | 9 | N/A |
| Highly satisfied (5) | 24 | 3 | 9 | 1 | 11 | N/A |
| **How does RECOVER compare to conventional neuroprognostication (mean)** | 4.45/5 | 4.22/5 | 4.75/5 | 4.5/5 | 4.35/5 | N/A |
| Not enough experience (NA) | 10 | 0 | 1 | 1 | 8 | N/A |
| Much Worse (1) | 0 | 0 | 0 | 0 | 0 | N/A |
| Slightly Worse (2) | 0 | 0 | 0 | 0 | 0 | N/A |
| Neither better nor worse (3) | 7 | 3 | 1 | 0 | 3 | N/A |
| Slightly Better (4) | 8 | 1 | 1 | 1 | 5 | N/A |
| Much Better (5) | 25 | 5 | 10 | 1 | 9 | N/A |

**Supplementary Table 2. Rated usefulness of neurology consultation for neuroprognostication (1 = Never useful; 2 = Rarely useful; 3 = Often useful; 4 = Always useful)**

|  | **Median (25%ile, 75%ile) consultation usefulness** |
| --- | --- |
| 2021-2022 neurologist physician (N = 52) | 3 (2, 3) |
| 2021-2022 non-neurologist physician (N = 63) | 3 (2, 3) |
| 2023 RECOVER naïve neurologist physician (N = 17) | 3 (2, 3) |
| 2023 RECOVER exposed neurologist physician (N = 9) | 3 (3, 3) |
| 2023 RECOVER naïve non-neurologist physician (N = 11) | 2 (2, 3) |
| 2023 RECOVER exposed non-neurologist physician (N = 13) | 3 (3, 4) |

**Supplementary Table 3. Rated frequency of comprehensive neuroprognostication (1 = Never; 2 = Rarely; 3 = Often; 4 = Always)**

|  | **Median (25%ile, 75%ile) frequency** |
| --- | --- |
| 2021-2022 neurologist physician (N = 52) | 3 (3, 3) |
| 2021-2022 non-neurologist physician (N = 63) | 3 (2, 3) |
| 2023 RECOVER naïve neurologist physician (N = 17) | 3 (2, 3) |
| 2023 RECOVER naïve non-neurologist physician (N = 11) | 3 (2, 3) |
| 2023 RECOVER exposed neurologist physician (N = 9) | 3 (3, 3) |
| 2023 RECOVER exposed non-neurologist physician (N = 13) | 3 (3, 3) |

**Supplementary Figure 1. Do you agree with the following statement? 'There is such limited evidence for the tests used in neuroprognostication that I do not see value in a neurology consult for neuroprognostication.'**

Respondents are divided into four groups: respondents to the 2021 and 2022 surveys (historical controls, combined for statistical analyses), respondents to the 2023 survey who reported exposure to the RECOVER program (RECOVER exposed) and respondents to the 2023 survey who denied exposure to the RECOVER program (RECOVER naïve [contemporary controls]). The Y axis represents the proportion of respondents within each group who provided each response. Error bars represent 95% confidence intervals.

**Supplementary Figure 2. In your experience, roughly how often is neurology consulted regarding neuroprognostication in comatose post-cardiac arrest patients?** Respondents are divided into four groups: respondents to the 2021 and 2022 surveys (historical controls, combined for statistical analyses), respondents to the 2023 survey who reported exposure to the RECOVER program (RECOVER exposed) and respondents to the 2023 survey who denied exposure to the RECOVER program (RECOVER naïve [contemporary controls]). The Y axis represents the proportion of respondents within each group who provided each response. Error bars represent 95% confidence intervals.

**Supplementary Figure 3. Do you agree with the following statement? 'Neurology consultation for neuroprognostication often leads to withdrawal of life-sustaining support in comatose post-cardiac arrest patients.'** Respondents are divided into four groups: respondents to the 2021 and 2022 surveys (historical controls, combined for statistical analyses), respondents to the 2023 survey who reported exposure to the RECOVER program (RECOVER exposed) and respondents to the 2023 survey who denied exposure to the RECOVER program (RECOVER naïve [contemporary controls]). The Y axis represents the proportion of respondents within each group who provided each response. Error bars represent 95% confidence intervals.

**Supplementary Figure 4. How much education have you received regarding neuro-prognostication in comatose post-cardiac arrest patients?** Respondents are divided into four groups: respondents to the 2021 and 2022 surveys (historical controls, combined for statistical analyses), respondents to the 2023 survey who reported exposure to the RECOVER program (RECOVER exposed) and respondents to the 2023 survey who denied exposure to the RECOVER program (RECOVER naïve [contemporary controls]). The Y axis represents the proportion of respondents within each group who provided each response. Error bars represent 95% confidence intervals. Trainees (residents and fellows) have been excluded.

**Supplementary Figure 5. How comfortable do you feel providing a neuroprognostic assessment for comatose patients after cardiac arrest?** Respondents are divided into four groups: respondents to the 2021 and 2022 surveys (historical controls, combined for statistical analyses), respondents to the 2023 survey who reported exposure to the RECOVER program (RECOVER exposed) and respondents to the 2023 survey who denied exposure to the RECOVER program (RECOVER naïve [contemporary controls]). The Y axis represents the proportion of respondents within each group who provided each response. Error bars represent 95% confidence intervals.

**Supplementary Figure 6. In comatose post-cardiac arrest patients who undergo targeted temperature management, when is neuro-prognostication ideally performed?** Respondents are divided into four groups: respondents to the 2021 and 2022 surveys (historical controls, combined for statistical analyses), respondents to the 2023 survey who reported exposure to the RECOVER program (RECOVER exposed) and respondents to the 2023 survey who denied exposure to the RECOVER program (RECOVER naïve [contemporary controls]). The Y axis represents the proportion of respondents within each group who provided each response. Error bars represent 95% confidence intervals.

**Supplementary Methods – Survey**

The goal of this survey is to review current practices surrounding neuro-prognostication after cardiac arrest across intensive care units at several hospitals within the University of Pennsylvania Health System. All responses to the survey will remain anonymous. Thank you for your participation!

1. What is your role on the patient care team?
   1. Nurse
   2. Advanced practice provider
   3. Resident
   4. Fellow
   5. Attending physician
2. How many years have you been in practice? OR Where are you in training?
   1. 0-5 a. intern
   2. 6-10 b. resident
   3. >10 c. fellow
3. (APP/PHYSICIAN ONLY) In what specialty did you train or are you currently training?
   1. Medicine/medicine subspecialty
   2. Neurology
   3. Anesthesia
   4. General Surgery/surgical subspecialty
4. At which hospital(s) do you work? Select all that apply.
   1. HUP
   2. PPMC
   3. PAH
5. How much education have you received regarding neuroprognostication in comatose post-cardiac arrest patients?
   1. None
   2. A little
   3. A fair amount
   4. A lot
6. In your experience, roughly how often is neurology consulted regarding neuro-prognostication in comatose post-cardiac arrest patients?
   1. Never
   2. Rarely
   3. Often
   4. Always
7. How useful is neurology consultation with respect to neuro-prognostication in comatose post-cardiac arrest patients?
   1. Never useful
   2. Rarely useful
   3. Often useful
   4. Always useful
8. In comatose post-cardiac arrest patients who undergo targeted temperature management, when is neuro-prognostication ideally performed?
   1. After 12 hours after rewarming
   2. After 24 hours after rewarming
   3. After 48 hours after rewarming
   4. After 72 hours after rewarming
9. In your experience, how often is a comprehensive neuroprognostic assessment performed on comatose post-cardiac arrest patients?
   1. Never
   2. Rarely
   3. Often
   4. Always
10. Do you agree with this statement: “Neurology consultation for neuro-prognostication often leads to withdrawal of life-sustaining support in post-cardiac arrest comatose patients.”
    1. Strongly disagree
    2. Somewhat disagree
    3. Somewhat agree
    4. Strongly agree
11. Do you agree with this statement: “Neurology consultation leads to a more comprehensive evaluation regarding neuro-prognostication in post-cardiac arrest patients.”
    1. Strongly disagree
    2. Somewhat disagree
    3. Somewhat agree
    4. Strongly agree
12. How comfortable do you feel providing a neuroprognostic assessment for comatose patients after cardiac arrest?
    1. Very uncomfortable
    2. Somewhat uncomfortable
    3. Somewhat comfortable
    4. Very uncomfortable
13. There is such limited evidence for the tests used in neuro-prognostication that I do not see value in a neurology consult for neuro-prognostication.
    1. Strongly disagree
    2. Somewhat disagree
    3. Somewhat agree
    4. Strongly agree

**The following questions were included only in the post-survey:**

1. Have you interfaced with the Recovery of Consciousness Via Evidence-Based Medicine and Research (RECOVER) Program (the neuroprognostication consult service at HUP) in providing clinical care? (Yes or No)
2. (If yes:) In what capacity have you interfaced with the RECOVER Program? (Can select multiple)
   - 1. As a member of the primary clinical team, requested a RECOVER consult
     2. As a member of the primary clinical team, collaborated with the RECOVER consult team in managing a patient
     3. As a member of the primary clinical team, collaborated with the RECOVER consult team in a family meeting
     4. Participated in the RECOVER team as a resident or fellow
     5. Joined interdisciplinary RECOVER conferences
     6. Other
3. (If yes:) How satisfied were you with the RECOVER service? (select one)
   - 1. 1 – Highly dissatisfied
     2. 2 – Somewhat dissatisfied
     3. 3 – Neither satisfied nor dissatisfied
     4. 4 – Somewhat satisfied
     5. 5 – Highly satisfied
4. (If yes:) How do you feel the RECOVER service (launched at HUP in August 2022) compares to conventional neurology consultations for neuroprognostication? (select one)
   - 1. 1 – The RECOVER service is much worse than conventional neuroprognostication
     2. 2 – The RECOVER service is slightly worse than conventional neuroprognostication
     3. 3 – The RECOVER service is neither better nor worse than conventional neuroprognostication
     4. 4 – The RECOVER service is slightly better than conventional neuroprognostication
     5. 5 – The RECOVER service is much better than conventional neuroprognostication
     6. N/A: I have not had sufficient experience to compare the RECOVER service with conventional neurology consultation
5. (If yes:) Please comment on any aspects of the RECOVER service that you like (responses are anonymous).
6. (If yes:) Please comment on any aspects of the RECOVER service that you do not like, or think could be improved for the future (responses are anonymous).

20) Did you complete the first iteration of this survey that was distributed in 2022?

- 1. Yes
  2. No
  3. I started but did not finish it
  4. I cannot remember
